# Applying a Complexity Lens to Policy Implementation: How Feedback Loops Help to Understand Systems Change in Integrated Healthcare

**DOI:** 10.1002/hpm.70140

**Published:** 2026-09-26

**Authors:** Andrew Passey, Peter van der Graaf

**Affiliations:** ^1^ School of Health Leeds Beckett University Leeds UK; ^2^ Department of Nursing Midwifery and Health Northumbria University Newcastle‐upon‐Tyne UK

**Keywords:** children's mental health, complexity, policy implementation and evaluation, schools, systems thinking, UK

## Abstract

**Background:**

Although a complexity theory lens can help to understand national policy implementation at a local government level, application of this lens often remains at a metaphorical level. We illustrate how complexity concepts, such as adaptation, feedback, emergence and co‐evolution can be used practically in research on policy implementation using a UK case study (Future in Mind; FiM), aimed at improving school children's access to mental health services in a metropolitan borough in the North England.

**Methods:**

We re‐analysed interview data with staff from local government, National Health Service/mental health, schools, and the voluntary and community sector (*n* = 31 semi‐structured interviews) involved in the implementation of FiM and coded this data in NVivo12 using complexity concepts as sensitising constructs.

**Findings:**

We identified 5 feedback loops: two positives (1. Flexing the training offer; 2. New skills, knowledge and behaviour by non‐specialist staff) and three negatives (3. Short termism, 4. Free rider behaviour, 5. Professional boundaries). These loops energised local adaptations of FiM by school and NHS staff, leading to system‐level change (emergence), with the school system becoming more responsive to mental wellbeing needs of children and young people, and shifts across systems (co‐evolution), by developing joint values and language between schools and NHS.

**Conclusions:**

We demonstrate the importance of positive and negative feedback loops for evidencing system‐level change and shifts across systems. Our findings provide new insights into unintended consequences of policy implementation and starting positions for implementers to utilise feedback for generating impact. We argue for a blurring of boundaries between implementation and evaluation in policy research to optimise the use of feedback.

## Highlights

- Use a complexity lens to understand unintended policy outcomes & chain reactions
- Blur boundaries between implementation and evaluation in policy research
- Identify feedback loops while implementing policy to timely adjust to co‐evolution
- Maximise positive feedback while minimise negative feedback in loops of learning

## 1 Introduction

Recent research in this journal and more widely has argued for local and national policy actors to embrace systems thinking as they implement public policy and view health care systems as complex system [1, 2, 3]. A complexity theory lens has been shown to enable novel insights on local implementation of nationally designed public policy [4, 5] with system archetypes helping to anticipate unintended consequences and factors influencing better utilisation [6]. However, some authors have been critical largely metaphorical and weak definition and operationalisation of key complexity concepts [7, 8, 9, 10] and point to the paucity of studies exploring how this can be achieved in everyday practice [11]. We present a practical application of complexity theory in research on policy implementation, which extends a ‘pragmatic view’ of reform processes [2] by arguing for a specific focus on feedback loops. As well as new empirical insights, we draw out wider implications for local actors working to implement public policy at the municipality level, including on the role of evaluation as a core component of implementation activities.

### 1.1 Applying a Complexity Lens

Complexity theory stems from an ontological view that the social world comprises complex systems, which have the capacity to self‐adapt in non‐linear and hence unpredictable ways. Small changes (e.g., a minor alteration to policy) can have disproportionately large effects, while seemingly large and consequential changes can lead to minimal shifts [12]. Key complexity concepts, such as feedback, self‐organisation, emergence and co‐evolution, have been seen as especially pertinent for studies of policy implementation [4, 13].

We specifically adopt an empirical focus on feedback in one defined system to map agent interactions, internal changes in the system, and changes at system level and between systems. As recent research has shown, a complexity lens that is rigorously conceptualised and applied in empirical research has potential to provide fresh insights on the implementation (and evaluation) of public health interventions [5]. Through a complexity lens, policy implementation can be viewed as an ‘event in a system’ with dynamic interactions between policy interventions and systems [14].

We apply this lens to a case study of the implementation of Future in Mind [15] (hereafter FiM) in Study‐borough (We use this as a pseudonym for a local authority area in the north of England, in which the research was undertaken). FiM was introduced across England in 2016, with five years of funding. The national aims of FiM were to improve children's access to mental health services and increase services delivered in schools, develop the mental health workforce, and reduce stigma associated with mental health issues. Study‐borough is a mixed urban and rural area in northern England with a population between 250,000 and 500,000. It was among the most deprived quartile of areas in England in 2015‐16 [16]. Local implementation of FiM involved the creation of a new multi‐organisation partnership comprising local National Health Service (NHS) commissioners and service providers, local government (municipality) services and local voluntary and community sector (VCS) (The National Council for Voluntary Organisations identifies six characteristics of organisations in the VCS: formal, independent, non‐profit distributing, self‐governing, having a meaningful degree of voluntary participation, a public benefit [https://www.ncvo.org.uk/news-and-insights/newsindex/uk-civil-society-almanac-2021/profile/voluntary-sector-definition/]) organisations. For the first time in Study‐borough, this approach brought together these organisations and clinical and non‐clinical staff to work with local children and young people.

Our adoption and application of a complexity‐informed lens to this case study is based on two critical justifications. First, the context in which FiM was launched was complex, with children's mental health and wellbeing proving a ‘wicked problem’ for policymakers [12, 17]. Evidence in the UK pointed to increased prevalence of mental health issues among children and young people, growth in need that was outstripping the provision of often fragmented services [18, 19]. Significant cuts in the UK to community‐based, preventative health and well‐being services, including but not only those focussed on children and young people, had potentially exacerbated the problem [20]. As argued by the guest editors of a special edition of Public Management Review on complexity theory, the ongoing existence of wicked policy problems points to a ‘need to continue to adopt and refine complexity‐informed theory, institutions, and practice in a domain of human endeavour as rich and varied as public administration’ [5]. In keeping with a complexity‐informed approach, we view ‘context’ as a separate but interacting system that possesses environmental, social, economic, cultural, physical, political, and technological dimensions. Examples of organisations in that wider context system include professional bodies, national lead agencies in and outside the NHS, pharmaceutical companies and policy organisations, all of which had potential for direct and indirect influence on FiM implementation.

Secondly, FiM is itself a complex policy response to a wicked problem. The national steer was that a broad range of organisations and professions from within and outside the NHS should be involved in implementing the policy [15]. Beyond this, the national policy was relatively loose and non‐prescriptive, which left space for local determination and room for adaptation by local partners based on local factors. These characteristics (a wicked policy problem and a relatively open policy response) suggest FiM as a ‘critical case’ [21] of complexity in implementation of public policy.

In this paper we draw on a case study [22], in which much of the data related to processes in the system, including those occurring in implementation of FiM. This orientation led us in our re‐analysis of these data to focus on feedback loops as mechanisms of change in the system, both to explore change within the system but also to potentially point to system‐level effects (emergence) and co‐evolution between systems, for example shifting system boundaries.

To operationalise our approach in this paper, we focus on three interconnected research questions.

1. What feedback loops are evident in the implementation of the national FiM programme in Study‐borough?
2. How do these feedback loops energise interactions between actors in the children and young people's mental health system to generate other features of complex systems (emergence, co‐evolution)?
3. What does this mean for local implementation of public policy interventions and research on their implementation?

## 2 Methodology

### 2.1 Data Collection

The original study on which this paper is based explored the extent and meaning of co‐production and partnership working for people involved in implementing FiM in Study‐borough [22]. All the participants in Study‐borough were directly engaged in implementation. They were drawn from across the range of organisations in the system through a stratified purposive sample design, a non‐random approach that involved identifying and recruiting individuals with important experience and knowledge for the original study [23]. Potential participants were contacted at meetings and other local forums where the lead author outlined the nature and scope of the research project and gauged potential interest in taking part. These contacts were subsequently invited by email to participate. Thirty‐one semi‐structured interviews were undertaken with local actors involved in implementing the policy. Four interviews were undertaken with NHS commissioners (Comm), seven with NHS Children and Adolescent Mental Health Services (hereafter CAMHS) managers and community‐based practitioners (CAM), nine with voluntary and community sector managers and community‐based practitioners (VCS), six with staff from the local authority, other service providers and regional‐level staff (LA/Other), and five with school‐based staff (Sch).

The lead author's host university granted ethical approval for the original study (Leeds Beckett University, granted on 10/07/2017). All participants in the original study were provided with written information prior to taking part in the research and gave their written informed consent to take part, including the use of quotes in written publications. We did not need any additional ethical clearance for the re‐analysis in this paper of anonymised interview data. Fieldwork lasted from September 2017 to January 2019 and all interviewees gave informed consent. Participants were asked about their work, with whom they worked, how much flexibility and autonomy they had; about their contact with children and young people as part of their role and what they had learnt through this work; about their understanding and views on a specific local aim to co‐produce services; and their views of, and experiences in co‐production and partnership working in the implementation of FiM in Study‐borough. Quotes are attributed to professional/sector groups, rather than individuals, to preserve anonymity while distinguishing different sectoral views.

### 2.2 Re‐Analysis of Existing Data

For this paper, we re‐analysed these data to reinterpret policy implementation through a complexity lens [24]. In an iterative process using NVivo software, we recoded interview data relating to features of cross‐organisation working in the partnership, workforce development, and sustainability of new ways of working. We began by defining and operationalising the complexity factors of interest to us, to provide a deductive analytical framework. To assess specific features of these factors in Study‐borough, our analytical process moved from general and broad deductive data (feedback) to specific feedback loops and their connections with one another and with other complexity factors.

We treated complexity concepts, such as adaptation, feedback, emergence and co‐evolution, as sensitising concepts [25]. Instead of looking for standardised and universal operational definitions for these concepts, we generated definitions that fitted the specific context with which the case study was interacting. To guide our coding, and to build cross‐coder consistency, we drew on pre‐existing definitions of key concepts of interest to us (Table 1).

**TABLE 1:** Context‐specific coding framework.

Concept	Sensitising definition	Themes (number of coded extracts per theme)
Adaptation	Changes made by staff in schools and CAMHS in response to the implementation of FiM in study‐borough (Skivington et al. [14]; HM Treasury [12]; Long et al. [26])	Organisational co‐production/delivery and governance; (19), Programme management (15)
Feedback loops	The result of interactions between stakeholders within the school and mental health systems in study‐borough when implementing the national future in mind (FiM) policy locally, which either negatively (diminishes/balances out) or positively (reinforces/promotes) other activities in both systems (Skivington et al. [14]; Cairney and Geyer [9]; Jalonen [27])	Positive feedback (19) 1.1. Adaptations to the training offer (6; F1) 1.2. Impact/benefits of upskilling for system (7) and other actors (6) (F2) Negative feedback (54) 2.1. Organisational resistance/inertia (23) 2.2. Financial uncertainty, context and short‐termism (15; F3) 2.3. Organisational barriers (14; F5) 2.4. Challenges to upskilling/free rider behaviour (2; F4)
Emergence	Changes at the system level arising from local‐level interactions between stakeholders in the education and mental health systems in study‐borough when implementing FiM (Mowles [28]; Skivington et al. [14]; Jalonen [27])	School responses to mental health issues (26) New ways of working in schools/more responsive to mental health of young people (16; E1) School reactions and concern to upskilling/consumerism (8; E2) Variation among schools in practices (17; E3)
Co‐evolution	New links between stakeholders, practices and values that emerged across system boundaries as a result of cross‐system interactions between stakeholders in the education and mental health systems in study‐borough when implementing FiM (Long et al. [26])	Joint values (as an epistemic shift) (4; C1) Joint language (as a system boundary shift) (4; C2)

### 2.3 Adaptation

We started by identifying adaptions from staff in schools and CAMHS in response to the implementation of FiM in Study‐borough. The relatively open nature of national FiM policy meant that new processes and system patterns in Study‐borough were locally derived responses to the policy [14]. These processes were evidence of system actors ‘learning or evolving, changing how the system behaves in response to interventions as they are applied’ [12]. Evidence of these responses, we coded as *adaptation*. For example, task groups were set up pre‐delivery between local commissioners and mental health providers to plan the implementation of FiM. This included new work with VCS organisations to design pathways and map existing services and changing governance structures overseeing delivery. Two main themes were coded under adaptation, covering (1) organisational co‐production delivery and governance; and (2) programme management, which included new multi‐agency forms of working.

### 2.4 Feedback

Following these adaptations, we looked for data on feedback. Feedback is the result of agent interactions, which might be stimulated through the introduction of a new policy in a system [12]. In a complex system, feedback occurs when ‘one change reinforces, promotes, balances or diminishes another’ [14]. Feedback might be direct or indirect, and ‘can work to both increase and accelerate or to suppress the changes taking place’ [12]. We defined amplification as positive feedback, and suppression as negative [9]. While positive feedback denotes instability and negative signifies stability, this does not imply a value‐based distinction of good or bad feedback [27], although different forms of feedback can have implications for the ‘success’ of policy implementation.

We coded data as *feedback* where there was evidence of agent interactions and processes that sought to promote and deepen the changes introduced in FiM, or where there was active or passive resistance to such changes. e.g., where participants commented on the positive benefits of upskilling staff, leading to staff being more responsive to mental health issues of young people in schools. Positive feedback mechanisms were evident in adaptation of workforce development processes, in increased skills and confidence among non‐specialists, and in changed behaviour by school staff. Negative feedback was manifest in limited engagement due to professional identities by some school staff in either the formal training programme and/or classroom‐based activities, and in limits to linkages between different professional groupings across Study‐borough. Feedback loops related to different perspectives on time. Positive feedback loops reflected a recognition among local actors of the urgency of the policy problem to which FiM was responding. In contrast, negative feedback tended to reflect concerns over the short‐term nature of the policy intervention.

Two main themes were coded under feedback: (1) positive feedback; and (2) negative feedback (see Table 1). There were two subthemes under positive feedback: adjusting of the training offer (F1); impact/benefits of upskilling for the system and other actors (relabelled new skills, knowledge and behaviour by non‐specialist staff, F2). Under the main theme of negative feedback, a further three subthemes were coded: financial uncertainty and short‐termism (relabelled short‐termism, F3); challenges to upskilling/free rider behaviour (relabelled free rider behaviour, F4); organisational barriers and context (relabelled professional boundaries, F5). We describe different positive and negative feedback mechanisms in more detail in our Findings section.

### 2.5 Emergence

Once we coded data into feedback loops, we explored what appeared to be consequent from them, a process that allowed us to derive and explore the specific parameters of other complexity factors (emergence, co‐evolution). Indications of system level change that arose from local‐level interactions we coded as *emergence*. Emergence is system level change that derives from agent interactions but is not simply reducible to them [28] and from locally driven responses and adaptations to an intervention [14]. We coded three themes under emergence: school responses to mental health issues, and new ways of working in schools/more responsive to mental health of young people (E1); school reactions and concern to upskilling/consumerism (E2); and variations among schools in practices (E3). Feedback mechanisms fostered, but did not fully account for, emergent processes at system level. Generally, the local system became more responsive to the mental wellbeing needs of children and young people, although new patterns of variation in practice emerged between schools. There was also evidence of a ‘consumerist’ orientation among staff in the sense that they were selective in the training they attended.

### 2.6 Co‐Evolution

As well as agent interactions, there is potential for systems to interact with one another and consequently to co‐evolve [26]. Since, in Study‐borough, FiM entailed new links across system boundaries, we were interested in evidence of cross‐system interaction and change. This we coded as *co‐evolution*. For example, co‐evolution between the mental health and school‐based education systems as school and CAMHS staff subscribed to joint values about mental health being everybody's business, giving them a deeper mutual understanding. We coded two themes under co‐evolution: joint values (as an epistemic shift) (C1); and joint language (as a system boundary shift) (C2). *Co‐evolution* manifested in some blurring of boundaries between the school education and children and young people's mental health services systems.

Our coding comprised work individually by each of the authors and working together in person or in video meetings. When we had completed individual recoding of relevant extracts of data as feedback, we compared and discussed the coding and made some minor changes, for example linking together different participant views on time, and identifying links between positive feedback loops. The latter included training and building the skills and confidence of school staff, which impacted on their behaviour. At this stage, we also relabelled feedback loops and identified two additional feedback loops ‘new skills, knowledge and behaviour by non‐specialist staff’ (F2) and ‘free rider behaviour’ (F4), while specifying links between loops and emerging system properties and co‐evolution. These changes were reflected in the explication of feedback in the system (see Table 2). Below, we discuss each feedback loop in turn, starting with positive loops, as well as associated evidence of emergence and co‐evolution. We do not discuss in detail adaptations as these are the starting point for interactions that create feedback loops, which is the main focus of paper.

**TABLE 2:** Summary of feedback loops.

	Feedback (F) loops		Other evident complexity factors
Positive feedback loops	F1 flexing the training offer	⟶	The overhang of the NPM was an evident *structural condition* in study‐borough, which manifested in consumerist behaviour by teachers and other non‐specialists (*emergent (E) property E2*)
	F2 New skills, knowledge and behaviour by non‐specialist staff	⟶	System became more responsive to the mental wellbeing needs of children and young people (*emergent property E1*). Teachers were less anxious labelling behaviour of children as a mental health issue, a change in their language that pointed to *co‐evolution (C) between systems (C2). Co‐evolution* between the mental health and school‐based education systems (*C1*) as school and CAMHS staff subscribed to joint values about mental health being everybody's business, giving them a deeper mutual understanding
Negative feedback loops	F3 short‐termism	⟶	The potential for the system to become more responsive to the mental wellbeing needs of children and young people *(emergent property E1)* was constrained by the limited time that mental health and VCS practitioners were able to spend in schools and with individual or groups of children and young people. This negative feedback was underscored by *structural conditions*: the urgent need to respond to the ‘policy problem’, the fixed‐term nature of FiM funding and shifting policy/funding attention
	F4 free rider behaviour	⟶	The required input of time from school staff reportedly did not always happen because of existing *structural conditions*, meaning that not every school provided this commitment. This manifested in myriad approaches in schools to children's emotional wellbeing (*emergent property E3*)
	F5 Professional boundaries	⟶	There was reported resistance to evolve professional identities among specialist staff, and among non‐specialist staff who reported concern about their limited expertise in children's mental health. This constrained the potential for *co‐evolution between systems (C1)*, underpinned by a *structural condition* of the system before introduction of FiM

## 3 Findings: Feedback Loops and How They Energise Features of Complex Systems (Emergence, Co‐Evolution)

At a national level, FiM included aims to develop the workforce and to increase school‐based services for children and young people [15]. The endogenous, locally driven approach to adaptation in Study‐borough involved linking these two national aims by developing the workforce in CAMHS and other staff working with children and young people in schools or in the VCS. The local focus was on community‐based services for children and young people of school age (between 5 and 18) experiencing mental distress at a ‘low level’ of acuity, an example of which might be that ‘a teacher who sees that a child is anxious, in a low mood, not eating or socialising as children and young people usually do, is withdrawn or behaving uncharacteristically, understands this child may need help’ [15]. The local programme included training for non‐specialists in the basics of child mental health and novel multi‐agency processes to better respond to individual cases of need. The term ‘non‐specialist’ is used to refer to staff working with children and young people on a day‐by‐day basis and so who were in scope of FiM but who were not employed by CAMHS. In addition, new community‐based roles in CAMHS and the VCS were funded by FiM to work across schools in Study‐borough and to coach and train non‐specialists ‘on the job’. Implementation was overseen and directed in Study‐borough by a new multi‐organisation partnership, exemplifying wider structural conditions in the UK oriented towards cross‐organisation and cross‐sector partnership, governance, trust, and greater accountability [29]. For example, the overhang of New Public Management (NPM) [30] was an evident structural condition in Study‐borough, which manifested in consumerist behaviour by teachers and other non‐specialists. In sum, these forms of adaptation fostered new interactions between agents, which directly and indirectly affected subsequent interactions through feedback loops in the system.

Table 2 summarises feedback loops in Study‐borough, their interactions with one another and with other complexity factors. Arrows are designed to illustrate linkages rather than denote causality given that emergence, for example, has been described as ‘a process in which things happen without clear and visible reason’ [27]. Further, the distinction between negative and positive feedback loops does not imply value judgements. Instead, this distinction delimits behaviour that suppressed change and stabilised pre‐existing features of the systems (negative feedback) from behaviour that amplified change (positive feedback). Implementors will be interested in understanding both, to help them and other actors adapt and maximise positive feedback while minimising negative feedback loops. A visualisation of the feedback loops in Table 2 can be found in Figure 1.

**FIGURE 1:**
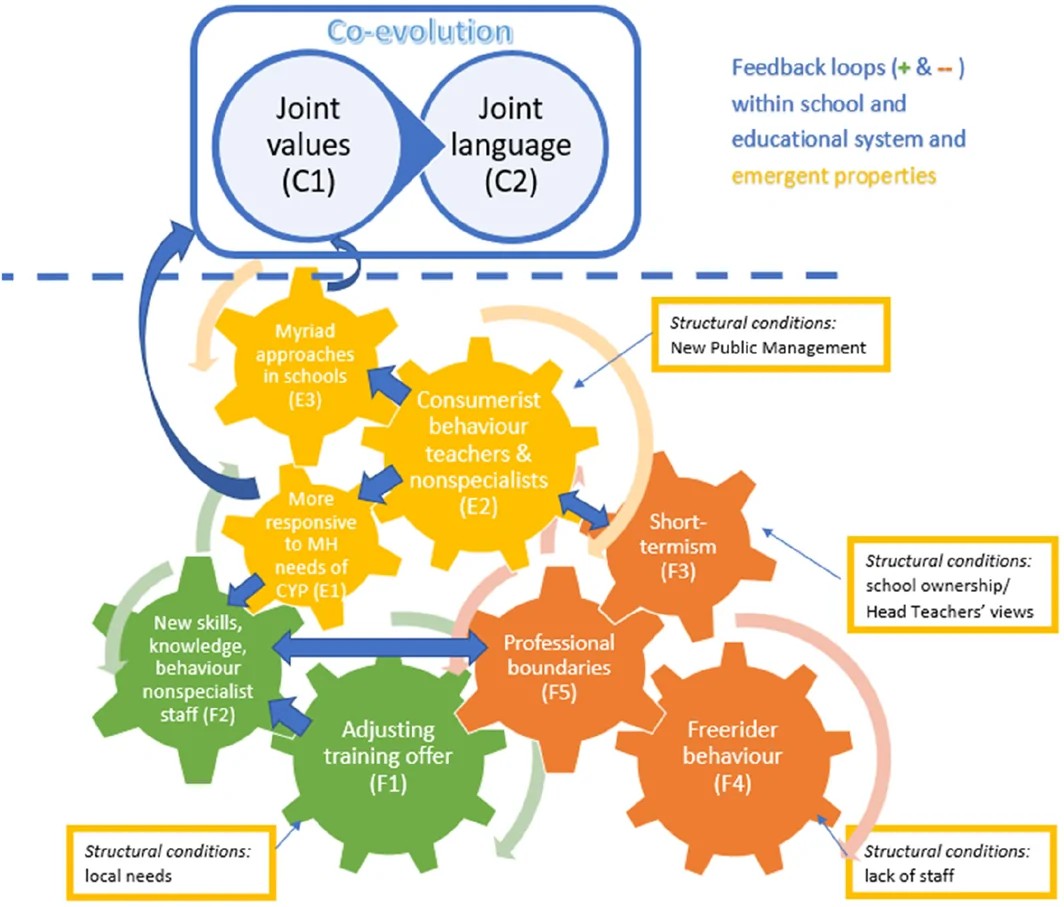
Visualisation of feedback loops, emergence and co‐evaluation in future in mind.

### 3.1 Positive Feedback

#### 3.1.1 F1 Adjusting the Training Offer

Interactions between agents (CAMHS, VCS staff, and teachers) generated positive feedback in adaptations to the training offer to better fit the local needs and preferences of non‐specialists, and through micro‐level agent interactions in schools during specific activities in the classroom. These changes were driven by non‐specialist staff and were seen to better enable their participation and upskilling. Training offered by CAMHS to non‐specialist staff represented a significant break with the past in Study‐borough. It was ‘offered out to everybody in Study‐borough working with children and young people’ [CAM], while previous training was only available to mental health professionals. However, despite the training being offered free of financial cost, CAMHS staff quickly realised that the requirement to sign‐up to a ten‐module programme was not feasible for school staff. Accordingly, CAMHS adapted its approach, so that participants could choose the topic modules they would attend. Agent interactions underpinned this change:

it was about just adjusting to school needs. And that was about listening to teachers, and what was appropriate and what wasn't appropriate for them, and what works. But obviously it wasn't just teachers, it was third sector [voluntary and community sector] services.

[CAM]

Adjusting was evident as well in micro‐level agent interactions in the classroom, with CAMHS staff reacting to issues identified by school staff. The kinds of issues school staff raised included children's ‘self‐esteem, resilience, sleep’ habits [CAM]. These issues could be specific to a single child or classroom, or might be common across a whole school:

We are kind of moving forward to a model of offering workshop‐based support to some staff in some schools, where they bring a theme that is coming up that they think is most relevant.

[CAM]

An unintended consequence of adapting the training offer was to encourage non‐specialist staff to adopt a selective approach to training by ‘just dipping into ones in which they're interested, so there isn't any continuity’ [LA/Other]. This *emergent property* (E2) limited the potential for non‐specialists to gain a broad grounding in mental health issues and techniques they might employ to engage with children and young people. It also challenged a local aim for cohorts of staff to learn together and forge new professional networks that might sustain once the training was completed. This experience in Study‐borough was suggestive of the New Public Management (NPM) reforms in public services ‘where consumer choice determines the best quality’ [30]. The overhang of the NPM was a *structural condition* in Study‐borough, which manifested in consumerist behaviour by teachers and other non‐specialists. Their needs dictated adaptation of the training offer, notwithstanding that the available ‘choice’ of content was constrained by specialist staff.

#### 3.1.2 F2 New Skills, Knowledge and Behaviour by Non‐Specialist Staff

Training provided to non‐specialist staff underpinned a second positive feedback loop, rooted in their acquisition of new skills and knowledge, and their shift to new ways of working. The purpose of the training was ‘it's about them having not in‐depth knowledge, but baseline knowledge and understanding, in terms of different aspects of children's mental health’ [CAM]. Actors from different organisations reported staff were ‘being upskilled massively …’ [CAM] and ‘seem to have benefitted from it, confidence wise a little bit’ [LA/Other]. The training was deliberately designed to ‘give people practical implementation for those things’ [CAM], such as ways to better engage with children and young people presenting with forms of emotional distress. New skills and knowledge manifested as feedback that energised change across schools in Study‐borough:

I think skills is possibly the right word. So we've done lots and lots of training, with the [CAMHS community‐based workers], and done lots and lots of work with schools to up‐skill them. So I think there a number of staff who have a desire to understand that and to embed that, that are in a better place.

[Comm]

In consequence of the training, school staff reportedly changed their views on children's mental health. The training was seen to be providing non‐specialists with a new view on issues like self‐harm, anxiety and depression that ‘if you've not got a mental health background, you think of in a different way, and understandably so’ [CAM]. Efforts to demystify some elements of specialist mental health knowledge meant that, to a degree, school staff began to internalise some of the perspectives of specialist staff. This was complemented in efforts by specialist staff and service commissioners to shift the mindset of schools, worked aimed towards ‘getting schools to realise that what we are trying to deliver is emotional health and wellbeing, is sort of a preventative rather than clinical model’ [Comm]. These activities underpinned a shift among actors from mental health being seen as a clinical issue to emotional wellbeing to a more social one, itself indicative of a system that was becoming more responsive to the mental wellbeing of children and young people (emergent property E1).

There was evidence of new behaviour by non‐specialist staff to support children presenting with emotional distress. This was closely linked with the provision and adjusting of training (F1) and new skills and knowledge among non‐specialist staff. School staff were reportedly less likely to immediately refer to CAMHS but would instead ‘hold’ the child in school and seek to engage with them and explore options to ameliorate their distress:

a lot of schools now … are more willing to hold young people at that emotional wellbeing level, you know very early stages of children presenting with self‐harm, where they would've probably directed straight into CAMHS.

[Comm]

One specific mechanism to effect this change was new multi‐agency working [31]. This adaptation in Study‐borough to the introduction of FiM included specific forums bringing together staff from the NHS, schools, local government and the local VCS. This was described as ‘people coming round the table and discussing things and getting some ideas together’ [Sch]. Multi‐agency forums helped to generate a richer picture of children and young people's experiences, as was evident in new information that bridged across systems in Study‐borough and which underpinned joint work ‘to look at what are the right support packages’ for specific children [Sch].

#### 3.1.3 Emergence and Co‐Evolution From Positive Feedback

Interactions between feedback loops energised adaptation in the system, which became more oriented to the needs of children and young people (emergent property E1). This shift was underpinned by shared values among staff across organisations about supporting children's emotional health and wellbeing. CAMHS staff and NHS commissioners successfully promoted to senior school staff and teachers that mental health was everybody's business and not just the responsibility of specialised clinical staff. This shared value base motivated teachers to engage with the training and look out for early warning signs to provide low‐level interventions to support children experiencing mental health issues. They could see how their actions would contribute to pupils' wellbeing, attendance, and ultimately their educational outcomes:

It was always about building sustainability within school, getting schools to realise that what we are trying to deliver is emotional health and wellbeing, is sort of [a] preventative rather than clinical model. So, it's everybody’s business within school, it's a core sort of element of what school can be providing to a young person […] so it's quite an easy sell.

[Comm]

Feedback, in the form of the training offer and its adaptations, manifested in an epistemic shift across systems, a form of *co‐evolution* between the mental health and school‐based education systems (C1). As both school and CAMHS staff subscribed to joint values about mental health being everybody's business, they gained a deeper mutual understanding. One example was the context in which schools were addressing mental health challenges, particularly in terms of limited resources:

As a CAMHS service [we] understand school pressure as well … my hope is that Future in Mind has helped, actually, the understanding on both sides.

[CAM]

The *Future in Mind* report [15] and the subsequent *Five Year Forward View for Mental Health* [32] had emphasised that the ‘vision’ for children's mental health and responsibility for the programme's ‘delivery’ was ‘everybody's business’. As such, the positive feedback loops evident in Study‐borough reflected that a specific *structural condition* prescribed in national policy discourse had been internalised by local implementers of FiM in Study‐borough (especially NHS commissioners and CAMHS staff).

Positive feedback also drove another form of *co‐evolution* between systems, energised by multi‐agency working rooted in new ways of working in response to the implementation of FiM. The presence and support for mental health in school settings became ‘just normal’ [VCS], a change in outlook across different sets of practitioners in their joint values and elements of a new joint language. Teachers were less frightened to label behaviour of children as a mental health issue, a change in their language that pointed to *co‐evolution* between systems (C2). One agent described how school staff had ‘started to use different language, use more kind of mental health language’, which they claimed reflected ‘a better understanding of perhaps what's happening for children’ [CAM]. Use of a broader language of mental health was viewed to mean, from a school perspective, that ‘you feel like you're automatically on the same page’ [Sch] with CAMHS colleagues.

Not all of this was a mutual shifting of boundaries between systems, but predominantly comprised school staff gravitating towards the language and epistemology of specialist mental health services. Although not evident after 2 years of Future in Mind in Study‐borough, one potential corollary is a drift towards the medicalisation of children and young people's emotional distress, involving a shift in emphasis from distress or difficulties to illness. Wider evidence of this in specialist mental services for children and young people has been the subject of critical review, grounded in a perceived shift towards acceptance of fixed, medicalised diagnoses of mental health and emotional distress, rather than a stance that argues for a greater focus on context. The authors argue that ‘[b]y conflating mental health with other health problems, we risk increasing medicalisation and further obscuring complex and intertwined family, community and socio‐economic contexts that produce and maintain distress’ [33].

### 3.2 Negative Feedback

Although the urgency of the policy problem underpinned recognition by participants of the need to actively respond, the limited time that mental health and VCS practitioners were able to spend in schools and with individual or groups of children and young people manifested in negative feedback (F3). Further, a *structural condition* (the fixed‐term nature of FiM funding) underscored negative feedback, with some school staff being less invested in the programme and CAMHS staff expressing worries that changes would not sustain once they withdrew from schools. There was also evidence of negative feedback in responses to efforts at workforce development, with some school staff failing to fully engage and adapt their own practice, resulting in free rider behaviour (F4) and the reported unwillingness of staff (CAHMS and school‐based) to cross professional boundaries (F5). Both these feedback loops were additionally fuelled by scepticism around the short‐term funding available for the programme.

#### 3.2.1 F3 Short‐Termism

The need to normalise mental health support in schools and develop new ways of working between school, CAMHS and VCS staff was fuelled by the high and growing numbers of children experiencing mental health issues. Local agents explained that the starting point for FiM was reduced spending compared with 2010 on local children and youth services, many of which had retrenched to focus on the most vulnerable families and away from preventative services. CAMHS reportedly tended to work in isolation in Study‐borough, was under pressure from long waiting lists for interventions, and was seen to lack the necessary capacity to support all of these children. In consequence, school staff required new skills and confidence to engage with these issues by themselves. As one CAMHS practitioner described it:

We're in an absolute crisis, which [..] is why Future in Mind was developed because we're in a pickle and children and young people need our help.

[CAM]

Worries about the short‐termism of responses to this urgent problem and concerns about the sustainability of FIM reflected a wider *structural condition* described as ‘a pocket of investment, then the investment moves on to something else’ meaning that ‘Future in Mind is [then] yesterday's news’ [CAM]. This condition dampened enthusiasm leading to a drag on system adaptation to the needs of children and young people (emergent property E1). In contrast to short‐term engagement, staff highlighted the need of continued investment of time in ‘revising, reviewing, reinforcing, learning, developing’ [CAM] mental health approaches at a system level to achieve impact. They pointed as well to a lack of understanding among policy makers and commissioners about the time involved in creating change within and between systems, ‘expecting this kind of miraculous change in a really short space of time’ [CAM]. Local actors were concerned that this would reduce the long‐term impact of the programme, despite initial positive changes and emerging behaviours being witnessed. This concern was fuelled by scepticism about the speed at which mental health challenges among children and their families could be resolved. Staff pointed to generational issues within families that required longer term attention and resources, with a focus on families and pre‐school age children:

I don't think we're going to see it for a really long time, possibly kind of another generation, because what I think we're working with is a generation of children where they're quite often being parented by people who have had issues in their own childhood and they've got mental health issues themselves, and perhaps can't prioritise that child's needs.

[CAM]

#### 3.2.2 F4 Free Rider Behaviour

Part of the workforce development package involved school staff shadowing staff from local VCS organisations, who were leading group work with primary school children on emotional resilience activities. VCS staff characterised these interactions as a ‘safe environment’ for school staff to see the work being delivered and to try it for themselves. This required input of time from school staff, which VCS staff reported did not always happen because of existing *structural conditions*, meaning that not every school provided this commitment: ‘some schools tried it on several times, because they're so busy and haven't got enough people to support, they would be trying to leave you on your own’ [VCS]. In effect, these agents were seeking to maximise their access to support services funded by FiM and delivered by the VCS, while downplaying their responsibilities to incorporate new practices or pass them on to other members of staff in the school, an approach that we view as containing elements of ‘free rider’ behaviour. Similarly, CAMHS reported that ‘our experience is kind of that a small number of the gold standard schools run with it and the rest still need reminding, handholding, encouraging’ [CAM]. This *emergent* variation (E3) was rooted in the *starting conditions* in the school‐based education system and pointed to the importance of path dependency in how the system changed [34]. In Study‐borough, a patchwork of school ownership and accountability manifested in myriad approaches in schools to children's emotional wellbeing. One corollary was that the attitudes of senior school staff were reportedly influential, with participants suggesting that ‘the Head Teacher kind of seems to dictate the thought process behind emotional wellbeing’ [CAM].

#### 3.2.3 F5 Professional Boundaries

A final negative feedback loop resulted from staff being reluctant to cross professional boundaries. This loop is linked to the positive feedback loop on new skills, knowledge and behaviour by non‐specialist staff (F2) and together they provide two distinct views on role perceptions. The positive feedback loop involved schools being more able and willing to hold children presenting with emotional distress, a change that was seen as rooted in the training school staff had received as part of FiM. A negative feedback loop was evident in reports by some school staff of feeling anxious about engaging with children on mental health issues, as they didn't see themselves as the experts and were continuing to expect CAMHS staff to come into schools solve their problems. Changing mindsets and normalising mental health support in schools by teachers, as described above, did not always occur, with some teachers actively resisting this change:

There has been a very mixed response really. [..] In the early days I think it was still very much “oh, we’ve got this problem, here over to you”. So, it has been quite a lot of work for the practitioners to actually say “well, yes, and what can you do?”.

[CAM]

Likewise, CAHMS staff were also resistant to giving up their professional identity in some instances. VCS staff complained that CAMHS workers sometimes stuck to their professional identity as a separate specialist service, not using the joint language and branding of ‘Future in Mind’. This made VCS staff feel less valued and feeling that they were perceived as amateurs who lacked the right qualifications, despite having (qualified) ex‐social workers in their teams:

It’s been very, very difficult to get the buy‐in from CAMHS. So even having a whole team away day, with CAMHS, [counselling service], and all the [VCS community‐based workers] talking about, you know, that they’re all working on this one project, this Future in Mind, CAMHS have maintained their “we’re CAMHS, we’re delivering a CAMHS service”.

[VCS]

FIM was partly commissioned to improve links between CAHMS and external organisations, however the reluctance of CAHMS staff to cross professional boundaries reduced collaborative working in FIM, as described by one local commissioner:

My plan, my big idea was to invest in primary intervention as a tool to fix CAMHS by osmosis … And what happened was I invested in something that got broken by CAMHS by osmosis.

[Comm]

This perspective suggests some continuation of prior views of CAMHS in Study‐borough, that it was inaccessible (‘sitting in their ivory towers’) and ‘a very, very traditional, old‐school type of service’ [Comm]. The potential for *co‐evolution* between systems (C1) was constrained by this negative feedback loop, underpinned by a *structural condition* of the system before introduction of FiM. One consequence was variation in co‐evolution intensity as agents adapted in response to the implementation of FiM.

## 4 Discussion: What This Study Means for Local Implementation of Public Policy Interventions and Their Evaluation

This paper contributes to complexity‐informed research on policy implementation. It extends a ‘pragmatic view’ of policy reform [2] in a practical empirical approach that emphasises the importance of feedback loops and raises implications for local policy actors and evaluators in municipality‐level public services. Our study of FiM exemplifies ‘a perspective on policy implementation as a self‐organising, emerging system in which local decision‐makers and public managers may pursue divergent policy options’ [2]. Our specific empirical focus enabled us to highlight unintended consequences and chains of reactions through feedback loops that affected not only the wider mental health system but also the educational system in Study‐borough. This resulted in co‐evolution—a systemic shift in mental health support with both schools and mental health provision becoming more responsive to the low‐level emotional wellbeing needs of local children and young people. It also resulted in a wider range of professionals engaging more directly, rather than referring children into already strained specialist services with long waiting lists. As well as suggesting a practical approach to revealing dynamism between complexity factors, we contribute to wider debates on the role of evaluators and implementers working to deliver and appraise public policy on the ground. We extend these points in two themes below.

### 4.1 Feedback Loops: Opening‐Up the Complex Dynamics of Policy Implementation

First, this paper moves beyond what has been criticised as the metaphorical use of complexity theory and concepts [9] to a more considered, but highly practical application that exposed wider system effects and dynamic linkages between complexity concepts. Our case study is reflective of a model in the implementation of public policy across a range of national contexts and policy issues [29] and provides a fresh lens on an often‐studied phenomenon [5]. We took that as one entry point and sought to contribute to understandings of policy implementation by strengthening the focus on feedback as the primary empirical lens. Through focussing on processes and mechanisms in the implementation of the policy (feedback), we generated evidence about other system properties (emergence and co‐evolution).

The Study‐borough case study revealed rich connections between positive and negative feedback loops, which interacted to generate emergent properties at system level and co‐evolution between systems. For example, the positive feedback loop of CAHMS adapting their training offer in response to local school needs (F1) energised new processes of engaging with and holding in school children presenting with emotional distress. Micro‐level agent interactions manifested in system effects, with the demands of non‐specialists increasingly shaping the work of professional mental health practitioners in the implementation of FiM. However, an unintended consequence occurred simultaneously, with teachers and other non‐specialist staff adopting a consumerist approach to training, which limited linkages between and learning opportunities for teachers. This emergent property (E2) reflected the overhang of New Public Management reforms in the educational system, which provided the starting conditions for it. The combined effect of this positive feedback and negative emergence was to embed variation among schools in the practice of mental health and wellbeing for young children (E3).

The shift in mental health support in Study‐borough suggested interactions between system‐level emergent effects and co‐evolution. In part, this emergent property was driven by co‐evolution between systems, with a degree of epistemic shift from specialist mental health professions into the views, language and understandings of children's mental health issues among school‐based staff. New agent interactions and shared language helped to generate a richer picture of children and young people's experiences, as was evident in new information that bridged across systems in Study‐borough. Although not all of this reflected a mutual shift of boundaries, it highlighted the importance of cross‐boundary understanding and the meaning‐making influence of terminology as it moves between systems.

### 4.2 Blurring Boundaries Between Policy Implementers and Evaluators

Second, our case study problematises prevalent distinctions between policy implementation and evaluation. This has potential to boost the steering capacity of local actors to open up implementation [2]. Applying a complexity lens highlights that agents, or teams of agents involved in implementing policy, need to act both as implementers and evaluators. This is akin to the boundary spanning roles suggested by Pelletier et al. [11] in this journal to support governance in multisectoral, complex adaptive systems. In effect, this involves bringing together an orientation towards complexity‐informed research [4] and a developmental evaluation approach [25]. It moves well beyond appraisal of the relative benefits of internal or external evaluation [35], to instead blur the boundary between implementation and evaluation. Such an orientation would adhere to several ‘principles’ of a developmental evaluation methodology, especially in relation to a focus on utilisation, co‐creation and timely feedback, systems thinking and a complexity perspective [25] that can be applied in different policy areas and municipalities. For example, evaluators focus on the nature and patterns of the intervention development to support adaptations and innovations, which are co‐created with implementors in an iterative process with timely feedback. In this collaborative process they are attentive to interrelations, boundaries and the context of the social system in which they are developing the intervention. To safeguard against bias and ensure sufficient critical distance, triangulation of data, reflective practice and periodic external audit is important. Applying developmental evaluation principles requires skills in collaboration, systems thinking and changes the way evaluators operate, with no clear, specific, and measurable outcomes that are to be achieved through processes detailed in a linear logic model up‐front, as is often the case in traditional evaluations. It also requires specific capacity (of roles and of time), which might vary from more typical approaches to evaluation.

Our empirical focus on mechanisms (feedback) highlighted emergent effects and change across systems, which might reveal evidence of the ‘success’, or not, of the policy implementation. In this more evaluative frame, positive and negative feedback might take on the additional meaning of being beneficial or not for the goals of the policy. We extend existing debates in this area, arguing for an orientation by those involved in implementing and evaluating public policy towards accepting and responding to complexity that reflects the notion of ‘complexification’ [2, 3]. This means that, instead of focussing on simplification, agents should apply ‘*a management approach … characterised by variety and diversity and a non‐mechanical way of working*’ [3]. Such an approach, the authors outline, requires broadening the scope of challenges and possible solutions, widening and reorientating structures (organisations and networks), and improvisation of processes. Following our case, this would involve identifying and evaluating feedback loops while implementing public policy and adjusting in response to emerging properties and co‐evolution processes at system level. This would, in effect, hybridise the work of policy implementation and evaluation.

For example, when evaluators encounter negative feedback loops, such as short‐termism, free‐rider behaviour, or professional boundaries they should not wait till the evaluation is completed to report their findings but discuss these emerging findings quickly with implementers to co‐produce adaptions that counter their destabilising effects. In our case study, this could have involved making cases for long‐term commitments from school leaders and long‐term funding from local commissioners to counter short‐termism; attaching commitments to funding for school staff to incorporate new practices in their school; working collaboratively with VCS organisations to counter free‐rider behaviour; and upskilling both school and CAHMS staff in cross‐boundary working to reduce anxiety and resistance. The key point is that feedback and co‐creation of adaptations needs to happen quickly to dampen the destabilising effect of negative feedback loops, while amplifying positive feedback loops.

For those responsible for policy ‘implementation’, adhering to this kind of approach would involve them acting as well as evaluators. This could include capturing evidence of different forms of feedback and feedback loops and focussing their efforts to maximise positive feedback aligning with the intent of the policy intervention, such as new forms of agent interaction as well as system‐level and cross‐system effects. Critically, actors would not ignore negative feedback, but instead accept it as inevitable and work to minimise it as the policy intervention plays out, based in a recognition that implementation of public health interventions is complex and ‘success’ requires loops of learning and adaptation to generate improved emergent outcomes. The corollary of this approach is to suggest ‘the way to achieve real improvement in outcomes is to enable the system to learn, improve and adapt’ [36].

The work of ‘researching’ and/or ‘evaluating’ policy implementation might include further refinement of complexity theory towards a practical, ‘pragmatic’ approach [2], more case studies (and we would argue cross‐case studies) to empirically explore interactions between complexity concepts as policy is being implemented [4] and ‘stories of employing complexity to unlock processes and achieve high‐quality results’ [3]. Studies like these could be enhanced by embedding researchers into implementation and delivery teams and services [37] or through new approaches to evaluation of ongoing public health interventions. One example is the PHIRST programme funded by the UK National Institute for Health and Care Research (NIHR Public Health Intervention Responsive Studies Teams (PHIRST) [https://phirst.nihr.ac.uk/about-phirst/accessed February 2, 2023]) where fully funded research teams with expertise in systems approaches are ready from the start of policy implementation to support evaluation of interventions in partnership and co‐design with local authorities. All these options have potential for co‐production of context‐sensitive and situated knowledge that accepts the complexity inherent to policy implementation, as well as formative learning processes and feedback loops. Each would need to be assessed by system actors to see if they are adding intended benefits and would require the building of skills for all involved [38].

### 4.3 Limitations of Our Study

There are some specific limitations in our approach that we discuss below in turn: (1) insights are drawn from a specific case study, (2) data was originally collected for a different purpose and re‐analysed for this paper, which (3) limits transferability of findings to other contexts.

Firstly, our findings are to an extent contingent upon our definition and demarcation of the system(s) of interest, and the specific case and the context with which it was interacting. We took a practical approach, given the difficulty of efforts to account for all effects across the whole of a system, but without viewing the system as substantial in the sense of it being fixed and unchanging. We were able to draw upon well‐established organisations, services and activities, and to an extent relationships, in delimiting the starting system into which FiM was inserted. The system at the introduction of FiM (its starting point) comprised NHS commissioners and service providers, specific local authority children and family services, along with children, young people and their families (service users/families of service users). This approach helped us identify those organisations that were more peripheral and enabled us to track changes in the system and between it and the contiguous school education system that was a focus of the particular local approach adopted in Study‐borough. This open and transparent approach aids the reader in assessing the case study methodology and findings, and the contribution of this paper.

Secondly, to an extent, the nature of the data, which were originally generated in a study of public service co‐production in policy implementation, dictated our deductive approach and emphasis on processes. Despite these acknowledged limitations, our re‐analysis of existing data added new insights to existing studies and illustrates that application of a complexity lens does not necessarily require new data to generate novel findings. Re‐reading of data is a strength of the approach in this paper.

Thirdly, our case study is set within an English context in one particular municipality and, therefore, different issues might apply in other countries with different governance and health systems. However, the literature suggests the ubiquity of these concerns. Across the globe, public health interventions are often complex, involve multiple actors and systems at different levels over longer periods of time, to which a complexity lens is well suited. The principal ways of working we have instilled from of our findings are transferable to other areas, as these ways of working are explicitly sensitive to different contexts.

## 5 Conclusion

The overarching conclusion is to build in a complexity‐informed lens from the start when designing public policy, implementing it, and researching or evaluating its implementation. One of our contributions is to highlight the potential of a focus on feedback as an effective means of case‐study research in complex systems. By identifying feedback mechanisms, both positive and negative, in response to adaptations made by local actors when implementing a public policy, we have explicated evidence of system‐level change (emergence) and shifts across systems (co‐evolution), which were energised by feedback. This provided new insights into unintended consequences of policy implementation and starting positions for implementers to utilise feedback for generating impact.

## Funding

The author has nothing to report.

## Conflicts of Interest

The authors declare no conflicts of interest.

## Data Availability

The data that support the findings of this study are available from the corresponding author upon reasonable request.
